# Harnessing the nursing and midwifery workforce to boost Australia's clinical research impact

**DOI:** 10.5694/mja2.51758

**Published:** 2022-11-06

**Authors:** Marion Eckert, Claire M Rickard, Deborah Forsythe, Kathleen Baird, Judith Finn, Andrea Gilkison, Richard Gray, Caroline SE Homer, Sandy Middleton, Stephen Neville, Lisa Whitehead, Greg R Sharplin, Samantha Keogh

**Affiliations:** ^1^ Rosemary Bryant AO Research Centre University of South Australia Adelaide SA; ^2^ UQ Centre for Clinical Research University of Queensland Brisbane QLD; ^3^ University of Technology Sydney Sydney NSW; ^4^ Curtin University Perth WA; ^5^ Auckland University of Technology Auckland New Zealand; ^6^ La Trobe University Melbourne VIC; ^7^ Burnet Institute Melbourne VIC; ^8^ Nursing Research Institute Australian Catholic University and St Vincent's Health Australia Sydney NSW; ^9^ Edith Cowan University Perth WA; ^10^ Centre for Healthcare Transformation Queensland University of Technology Brisbane QLD

**Keywords:** Ethics, research, Clinical trials as topic

The largest health workforce has the greatest research potential; investing in nursing and midwifery researchers is an investment in better care and cost outcomes

For the Medical Research Future Fund (MRFF) to achieve its full impact, it is necessary for health practitioners to be trained and reliably funded to deliver research and translation alongside their clinical work.^1^ We offer insight into current systems, concerns and suggestions as this applies to clinical research in nursing and midwifery.

Nurses and midwives globally have a long record of delivering high quality clinical research that improves care and outcomes. An analysis of four landmark nursing‐led studies in the United States illustrates the value‐adding potential of such research: for every grant dollar, the return on investment ranged from $202 to $1206.^2^ In Australia, investment in nursing‐ and midwifery‐led research also pays dividends for health care costs and population and health system outcomes, as evidenced from the many research contributions of Australian nurses and midwives over the past decade (Box).^3^, ^4^, ^5^, ^6^, ^7^, ^8^, ^9^, ^10^

Box 1 **Select contributions from nursing‐ and midwifery‐led research in Australia**

**Important care outcomes**

- Decreased incidence of catheter‐associated urinary tract infections through use of chlorhexidine for cleaning before urinary catheterisation^3^
- Reduced short term death and disability and longer term mortality from stroke nursing protocols^4^
- Reduced psychological distress and increased preparedness for caregivers of palliative care patients^5^
- Effective relief from labour‐related pain with water injections^6^

**Major savings for the health system**

- Less frequent replacement of infusion sets shown to be safe, reducing national costs by $75 million annually for central venous and peripheral arterial catheters^7^
- Reduced health care costs ($13 100 less per person) and duration of all‐cause hospital stay (10 days fewer) with home‐based intervention for older patients with chronic heart failure^8^
- Caseload midwifery proven safe and cost‐effective for women of any risk, saving more than $560 per woman^9^
- $65 million savings in health care costs and $252 million savings in societal costs from stroke nursing protocols over 5 years^10^

Nurses and midwives are the frontline workers in hospitals and communities and thus are well positioned to lead research addressing efficacy of clinical and health system interventions. Nurses and midwives work across all aspects of health care delivery, across all age groups, and from metropolitan to rural and remote areas, making their reach and potential impact substantial. To achieve meaningful and sustained impacts on health care outcomes, greater engagement with, and investment in, nursing‐ and midwifery‐led research is needed.

## The largest health workforce is underrepresented as recipients of research funding

Health care is a $181 billion dollar industry in Australia,^11^ and care based on high quality evidence is essential to the health of the nation. Clinical trials and clinician researchers play a vital role in improving the health care system to benefit consumers, society and the economy.^12^, ^13^ The National Health and Medical Research Council (NHMRC) and the MRFF aim to invest in research informed health care, underpinned by transformative and innovative studies conducted by a skilled and capable health and medical research workforce.^14^, ^15^ However, analysis of research funding reveals profound inequity in the distribution of funds.^16^

In Australia, there are over 479 000 nurses and midwives serving our communities.^17^ Nurses and midwives constitute 57% of registered health professionals, making them the largest group in the workforce.^18^ Nursing and midwifery interventions are high volume and significantly contribute to both costs and patient outcomes, so the need for a strong evidence base is clear. Although Australia's nurses and midwives are ideally placed to provide solutions to current health service inefficiencies, they are underrepresented as recipients of research grant funding. For example, of the 200 NHMRC grants funded to clinical trials networks between 2004 and 2014, only nine (5%) involved nursing and midwifery‐specific research;^12^ in 2020, the NHMRC Investigator Grants scheme saw only seven of 238 grants (3%) awarded to nursing and one to midwifery (0.4%);^16^ and only one NHMRC 2020 postgraduate scholarship was awarded in nursing (1.6%).^16^ Notably, of all NHMRC 2020 grant round applications, only five of 673 successful applications (0.74%) were nursing or midwifery focused, and only 30 of 5221 total applications (0.57%) identified nursing or midwifery as the primary field of research.^19^ A severe lack of nursing and midwifery applicants is a major issue.

The nursing‐ and midwifery‐led research space is also likely disproportionately affected by gender disparities in grant outcomes, owing to the high percentage of female nurses and midwives in Australia (over 88%).^17^ The NHMRC grant success rate for mid‐career women is only 6.5% (compared with 10.9% for men).^20^ In 2021, women received 23% fewer NHMRC grants and were awarded $95 million less in funding compared with men.^21^ As identified in the CEO Communique in February 2022 on gender disparities in the NHMRC Investigator Grants scheme, the proportion of female applicants each year also declines quickly with seniority.^22^

## Employment structures affect ability to lead research that improves the health system

Grant criteria have also inhibited nurses’ and midwives’ opportunities to apply for funding. For example, the 2020 MRFF Clinician Researchers initiative grants required chief investigators to be clinician researchers (defined as health professionals who practised in a clinical capacity).^23^ Historically, nurses and midwives have had to choose between a clinical or academic career, partly because of the way in which nursing and midwifery care is provided. Academic nurses or midwives and those employed by health sectors as independent researchers rarely deliver direct clinical care; juxtaposed with clinical nurses and midwives who typically have positions without any included seconded or protected research time. Thus, until now, the narrow definition of clinician researcher excluded many nurses and midwives.

The Australasian Nursing and Midwifery Clinical Trials Network, a consortium of senior nursing and midwifery academics across Australia and New Zealand, was recently established to support nurses and midwives to undertake high quality research. The Australasian Nursing and Midwifery Clinical Trials Network liaised with the MRFF following the announcement of the 2020 grant round, and advocated for change to the definition of clinician researcher to ensure inclusivity for nurses and midwives. Whether directly or indirectly related to this advocacy, the definitions under the latest scheme have addressed this, with the 2022 Clinician Researchers: Nurses, Midwives and Allied Health Grant Opportunity guidelines defining a clinician researcher as “a researcher that has current professional registration with the Australian Health Practitioner Regulation Agency” (unpublished document, National Health and Medical Research Council, 2022). In addition, Stream 1 is exclusively for research led by a nurse or midwife. This is an important and welcome first step towards equity for nursing and midwifery researchers.

Nevertheless, the issue is broader than grant scheme eligibility. Lack of protected research time for clinicians restricts their ability to write grant applications, and in the event of winning a grant, presents concerns around work–life balance for those undertaking both research and clinical work. Clinicians and managers may also need practical support to assist with applying for research grants, and there is need for more researchers across the nursing and midwifery workforce who are adequately trained and skilled to design and lead high quality clinical research. Tapping into the great potential of the nursing and midwifery workforce requires building the research capacity and capability of the workforce, such as by supporting early and mid career research fellowships as in other disciplines, and by strengthening undergraduate research training.

Although the end goal of greater investment in nursing‐ and midwifery‐led research is to improve health care outcomes, it is possible that a push for more researchers could further exacerbate clinical nursing and midwifery shortages. It is important that we work to boost the whole nursing and midwifery workforce, and integrate research as part of the roles, not simply transform our clinicians into researchers.

## Moving forward

Much of the work needed to boost nursing‐ and midwifery‐led research in Australia should be led by those in nursing and midwifery leadership. However, action is required more broadly to ensure transdisciplinary policies and initiatives for research training opportunities, funding and systems.

For nurses and midwives, strategies are needed to:

- develop research skills:
  ▶ by further improving the teaching of undergraduate level research skills and enabling conversion to honours programs;
  ▶ by bolstering doctoral and postdoctoral research training opportunities and ensuring suitability of programs for nurses and midwives, including those who remain clinically active; and
  ▶ by improving the quality of nursing and midwifery research outputs; and
- increase resources:
  ▶ by funding opportunities and embedding career frameworks for nurses and midwives to undertake research that is clinically embedded, whether or not they undertake direct clinical work; and
  ▶ by creating nursing and midwifery roles that are part clinical and part research, and providing clinicians with dedicated time alongside their care duties to undertake clinical research and translation work (akin to medical colleagues).

Research funding and opportunities should be sustainable, equitable, efficient and responsive to clinical needs.^24^ Inequalities in research funding across gender and discipline divides should be considered by government and funding bodies when creating funding priorities and grant criteria. We look forward to seeing how recent changes may begin bridging these divides. Nurses and midwives comprise most of Australia's regulated health care workers. They should therefore be key players in the design, development and leadership of clinical research, and their support as future research leaders is a sound economic investment.

## Open access

Open access publishing facilitated by University of South Australia, as part of the Wiley ‐ University of South Australia agreement via the Council of Australian University Librarians.

## Competing interests

No relevant disclosures.

## Provenance

Not commissioned; externally peer reviewed.
